# Attitudes toward Euthanasia of Students of Law and Medicine in Serbia

**Published:** 2020-02

**Authors:** Darko DIMOVSKI, Veljko TURANJANIN, Milica KOLAKOVIĆ-BOJOVIĆ, Dragana ČVOROVIĆ

**Affiliations:** 1. Faculty of Law, University of Niš, Niš, Republic of Serbia; 2. Faculty of Law, University of Kragujevac, Kragujevac, Republic of Serbia; 3. Institute of Criminologial and Sociological Research, Belgrade, Republic of Serbia; 4. Academy of Criminalistic and Police Studies, Belgrade, Republic of Serbia

## Dear Editor-in-Chief

Who is the owner of human life and body? Human civilization is trying to answer this question for centuries, but without great success. These are seemingly philosophical questions; however, responses can have quite practical consequences (1). If a person who is in the terminal phase, in a certain situation wish to end his/her life, this can happen with the active or passive role of a physician. In the Republic of Serbia, as the country in which euthanasia (hereinafter: VAE) is prescribed as a criminal offense (2), there have not been many empirical types of research in relation to the attitudes of the population about VAE.

In a certain way, our research is a continuation of the research conducted among medical staff and citizens (3–5). There is a good reason for medical and law students were chosen as the research sample. Namely, students of the law faculties are future decision-makers. Medical faculty students are relevant as s research sample having in mind their future role in situations where their patients are in the terminal phase of the disease, and they should make decision on termination of their patients’ lives. Having in mind earlier studies on students’ opinion regarding this issue, students of medicine were mostly the research target group while law student was out of research focus (6–8).

We conducted a survey among students in Kragujevac and Niš (Serbia) in the second half of 2017. The research included 214 respondents, with an equal number of respondents from law faculties and medical faculties - 107.

All participants in this study expressed the consent to participate and they returned completed questionnaires in the closed envelopes.

The questions from the survey were as follows (Table 1):

**Q1**. Do you think that the physician should end the life of a patient who asked him of to deprive him of life because of the patient’s severe health condition?
**Q2**. If a patient suffers from a disease that will inevitably ruin his mental or physical health, and wants to take his own life, but he cannot do it alone, the physician should be allowed to use VAE.
**Q3**. Patients should have the option of requiring VAE when they are faced with an incurable disease that is in the terminal phase?
**Q4**. Whether a physician should be allowed for VAE over a patient who is 24 yr old and a victim of a fire resulting in severe pain and requires VAE?
**Q5**. The patient suffers from cancer, which causes him unbearable pain, not alleviated by medication. The patient requesting the use of VAE. The physician should be allowed to inject intravenously a dose of a medicine that will cause death of the patient.
**Q6**. In case of VAE legalization, would you, as a patient suffering from a severe incurable disease, request VAE of yourself to be executed?
**Q7.** Under what circumstances would you, as a patient suffering from a severe incurable disease, require VAE to be executed in case of its legalization?
**Q8**. Would you vote in the referendum on VAE legalization?
**Q9**. VAE is ethically acceptable?
**Q10**. I support the legalization of VAE for all age groups.

**Table 1:** Results obtained from the questions of the survey

*No. of question*		*Results*		
Q1				
Yes	No			
54%	46%			
Q2				
Completely agree	Partially agree	Mainly agree	Mainly disagree	Completely disagree
18.1%	31.5%	15.7%	16.7%	18.1%
Q3				
Completely agree	Partially agree	Mainly agree	Mainly disagree	Completely disagree
27.3%	27.3%	19.4%	12.5%	13.4%
Q4				
Completely agree	Partially agree	Mainly agree	Mainly disagree	Completely disagree
7%	20%	8.4%	24.7%	40%
Q5				
Completely agree	Partially agree	Mainly agree	Mainly disagree	Completely disagree
22.2%	25.5%	19.4%	17.6%	15.3%
Q6				
Yes	I do not know		No	
18.6%	57.2%		24.2%	
Q8				
Yes	No			
62.4%	37.6%			
Q9				
Completely agree	Partially agree	Mainly agree	Mainly disagree	Completely disagree
13.3%	31.7%	19.3%	18.3%	17.4%
Q10				
Completely agree	Partially agree	Mainly agree	Mainly disagree	Completely disagree
10.1%	18.3%	16.1%	22.5%	33%

Among students who answered at Question 7, some of frequently given explanations were related to terminal phase of an incurable disease; unbearable pain; amnesia; autoimmune disease; disability to deal with dally routine activities; loneliness; if the disease diminishes the quality of life of the rest of the family and patient became a burden to people in his environment; deep ageold; medication resistance; degradation of the psychological condition and mental illness.

The results of this research show the complexity of this issue, but also the need for further implementation, whereby the state must take into account the citizens’ majority views on euthanasia.

Whether or not it is a question of granting euthanasia, it is necessary to come very carefully, taking into account all aspects of this problem, while respecting the views of the public opinion.
